# Highly Biaxially Strained Silicene on Au(111)

**DOI:** 10.1021/acs.jpcc.0c11033

**Published:** 2021-05-04

**Authors:** Daniele Nazzari, Jakob Genser, Viktoria Ritter, Ole Bethge, Emmerich Bertagnolli, Georg Ramer, Bernhard Lendl, Kenji Watanabe, Takashi Taniguchi, Riccardo Rurali, Miroslav Kolíbal, Alois Lugstein

**Affiliations:** † Institute of Solid State Electronics, Technische Universität Wien, Gußhausstraße 25-25a, 1040 Vienna, Austria; ‡ Infineon Technologies Austria AG, Siemensstraße 2, 9500 Villach, Austria; § Institute of Chemical Technologies and Analytics, Technische Universität Wien, Getreidemarkt 9, 1060 Vienna, Austria; ∥ Research Center for Functional Materials, National Institute for Materials Science, 1-1 Namiki, Tsukuba 305-0044, Japan; ⊥ International Center for Materials Nanoarchitectonics, National Institute for Materials Science, 1-1 Namiki, Tsukuba 305-0044, Japan; # Institut de Ciència de Materials de Barcelona, ICMAB-CSIC, Campus UAB, 08193 Bellaterra, Spain; % Institute of Physical Engineering, Brno University of Technology, Technická 2, 616 69 Brno, Czech Republic; & CEITEC BUT, Brno University of Technology, Purkyňova 123, 612 00 Brno, Czech Republic

## Abstract

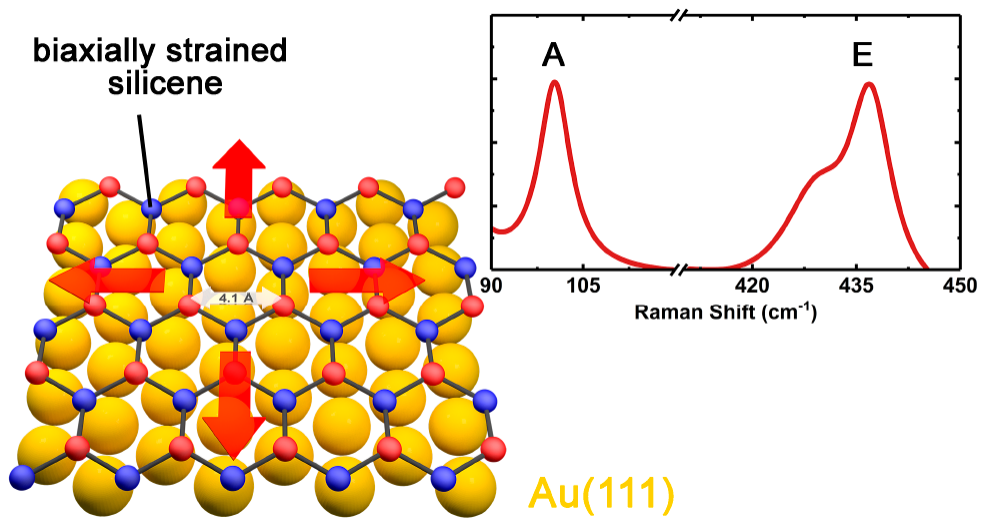

Many of graphene’s remarkable properties arise from its linear dispersion of the electronic states, forming a Dirac cone at the K points of the Brillouin zone. Silicene, the 2D allotrope of silicon, is also predicted to show a similar electronic band structure, with the addition of a tunable bandgap, induced by spin–orbit coupling. Because of these outstanding electronic properties, silicene is considered as a promising building block for next-generation electronic devices. Recently, it has been shown that silicene grown on Au(111) still possesses a Dirac cone, despite the interaction with the substrate. Here, to fully characterize the structure of this 2D material, we investigate the vibrational spectrum of a monolayer silicene grown on Au(111) by polarized Raman spectroscopy. To enable a detailed *ex situ* investigation, we passivated the silicene on Au(111) by encapsulating it under few layers hBN or graphene flakes. The observed spectrum is characterized by vibrational modes that are strongly red-shifted with respect to the ones expected for freestanding silicene. By comparing low-energy electron diffraction (LEED) patterns and Raman results with first-principles calculations, we show that the vibrational modes indicate a highly (>7%) biaxially strained silicene phase.

## 1 Introduction

Silicene, the graphene-like 2D allotrope of silicon, has attracted great attention over the past few years because of its predicted outstanding electronic properties.^1−3^ Theoretical studies of the band structure of freestanding silicene have described the presence of a Dirac cone,^4^ similarly to what can be observed in graphene.^5^ Additionally, spin–orbit coupling induces the opening of a bandgap that can be tuned via the application of an external electric field.^6,7^

Experimentally, silicene has been successfully grown by molecular beam epitaxy on different crystalline substrates.^8−10^ Most of the experimental studies have been realized by using Ag(111) as a substrate. Early experimental observations of the silicene/Ag(111) structure have reported signatures of a Dirac cone, despite a non-negligible interaction with the substrate.^11,12^ Subsequent studies have, however, pointed to an absence of such cone,^13−15^ showing that the interaction with Ag(111) alters the electronic properties of the 2D silicon layer.

Recently, Au(111) has been explored as an alternative viable substrate for the growth of silicene with preserved Dirac Fermions. High-resolution angle-resolved photoemission spectroscopy experiments support the presence of a Dirac cone and a bandgap of ≈0.5 eV, with an estimated Fermi velocity of 10^6^ ms^–1^, similar to the one of graphene.^16^ Moreover, scanning tunneling microscopy experiments demonstrated that the 2D silicon layer grown on top of Au(111) can be easily removed by applying a small voltage pulse of 3 V, implying a rather weak interaction with the substrate.^17^ At the same time, however, others reported the formation of a highly biaxially strained silicene phase, characterized by a lattice constant of 4.1 Å, indicating non-negligible interaction between substrate and epitaxial layer,^18^ stronger than originally assumed.

Here, we report the investigation of the structural properties of 1 monolayer (ML) silicene grown on Au(111) by micro-low electron energy diffraction (μ-LEED) and polarized Raman spectroscopy, enabled by an *in situ* passivation using few layers hBN or graphene flakes. On the basis of symmetry considerations, we can clearly assign the observed spectrum to a 2D silicon structure. By comparing the obtained LEED and Raman spectra with first-principles calculations, we show that silicene grown on Au(111) is highly biaxially strained (>7%).

## 2 Experimental Details

Silicene on Au(111)/mica (MaTecK) is grown in a custom-built UHV system (TU Vienna) at a base pressure of 5 × 10^–11^ mbar. The substrate is cleaned through three cycles of Ar^+^ ion sputtering (1 keV, 5 min), followed by annealing at 770 K for 15 min. One monolayer (ML) of silicon is evaporated from a rod by electron beam evaporation (EBE-1, SPECS) with a deposition rate of ≈0.02 ML/min, while keeping the substrate temperature at 533 K. The temperature is controlled by an infrared pyrometer (DGE-10N, DIAS; spectral range 2–2.6 μm) with a precision of ±2 K.

Microdiffraction analysis is performed by using a low-energy electron microscope (LEEM, SPECS FE-LEEM P90), part of a complex UHV system at CEITEC, Brno University of Technology, where the samples were prepared in the same way as described above. To perform diffraction in LEEM, electrons are extracted from the field emission source and accelerated to 15 keV. After passing the electron optics, they are decelerated by a strong electric field in the vicinity of the sample to very low energies (0–40 eV). The diffraction patterns are collected by using 30 eV electrons from areas selected by microdiffraction aperture (spot size ranging from 15 μm down to 185 nm).

To enable detailed polarized micro-Raman characterization, silicene was passivated *in situ* immediately after the growth by mechanical exfoliation of few-layer hBN or graphene flakes (HQ Graphene)^19^ in a dedicated UHV chamber, directly connected to the evaporation chamber.

*Ex situ* Raman analysis is performed in a backscattering geometry by using two different confocal micro-Raman setups. The first is an Alpha300 (WITec) stage equipped with a 532 nm (2.33 eV) Nd:YAG laser and a grating monochromator with 1800 grooves/mm. The laser is focused onto the sample by a Nikon Epiplan 100× objective, enabling a spot size of ≈700 nm. The second setup (Alpha500, WITec) is capable of detecting vibrational modes with a frequency as low as 75 cm^–1^ and is equipped with a grating monochromator with 1200 grooves/mm and a 532 nm (2.33 eV) Nd:YAG laser, focused by a Zeiss EC Epiplan-Neofluar 100× objective down to a spot size of ≈800 nm. All measurements were performed under ambient conditions, and moderate laser power, to avoid heating effects.

The samples analyzed by LEEM and Raman spectroscopy are grown by using the same process as well as employing the same substrates and the same evaporation material. To further confirm that the samples are identical, the ones grown in Vienna are further analyzed by using LEED (ErLEED 1000A, Specs).

Density-functional calculations are performed with the ABINIT code^20^ to obtain the ground-state geometry in different strain configurations to compute the phonon modes at the Γ point and the corresponding Raman susceptibility tensors.^21,22^ We used norm-conserving pseudopotentials, a plane-wave cutoff of 48 hartrees, the local density approximation for the exchange-correlation energy, and a 22 × 22 **k**-point grid to sample the Brillouin zone. Further details about the Raman calculations can be found, for example, in ref (23).

## 3 Results and Discussion

**Figure 1:**
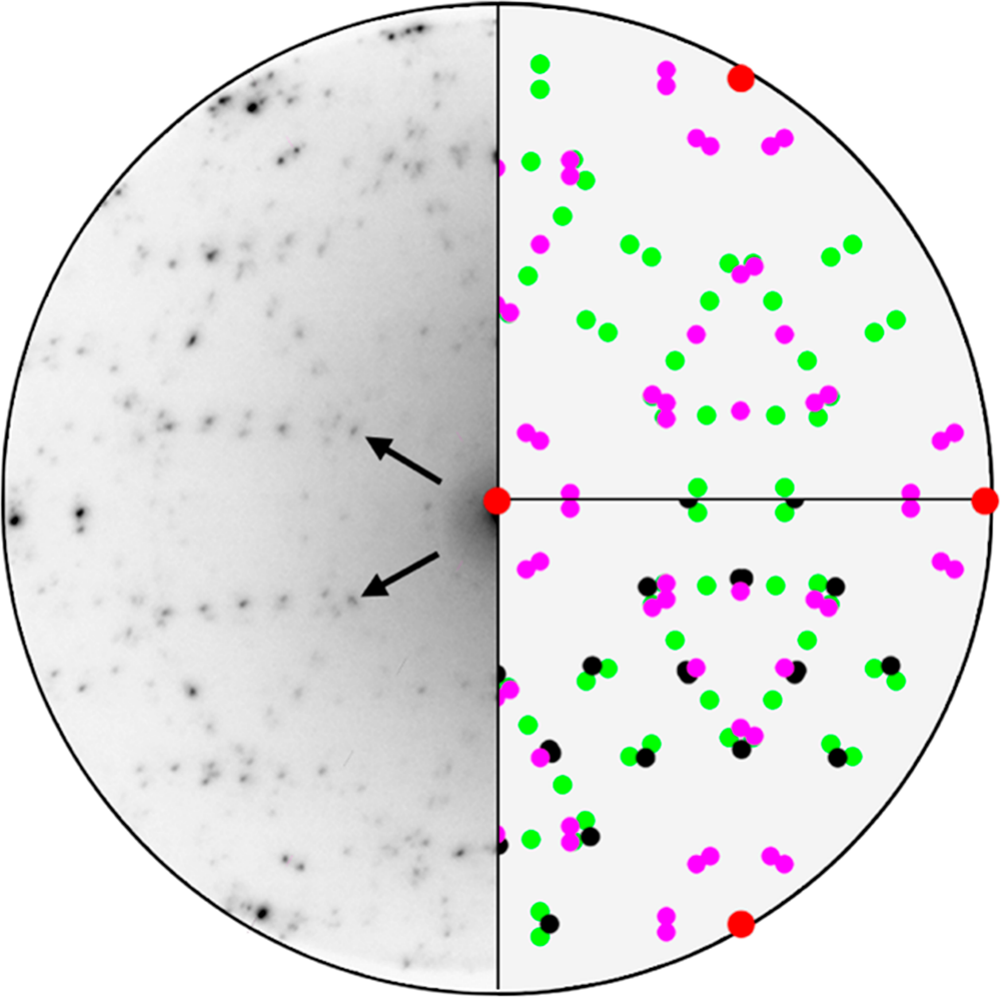
Microdiffraction pattern (left half of the image) of the grown layer, acquired at an electron energy of 30 eV. Two different diffraction models are presented in the right half of the image. The red dots represent the diffracted beam from the Au(111) substrate. The upper right quarter model assumes the presence of two surface silicide phases (green and purple). Some of the brightest unexplained dots of the microdiffraction pattern (indicated by the arrows) are only accounted for by a silicene-derived phase (black), as shown in the lower right quarter.

Immediately after the cleaning of the Au(111) surface, 1 ML of silicon was evaporated at a substrate temperature of 533 K. Subsequently, a microdiffraction image was collected at an electron energy of 30 eV, as shown in Figure 1.

A well-reproducible, complex diffraction pattern was observed alongside the signal associated with the Au(111) substrate (red dots in Figure 1). The pattern cannot be explained by a simple single-superstructure model, implying the presence of several mixed phases. The appearance of a multiphase diffraction pattern is not surprising: in a similar system—1 ML of silicon on Ag(111)—the diffraction pattern is always reflecting the presence of multiple silicene phases due to the very similar formation energies.^24,25^ The very same microdiffraction pattern was detected all over the substrate, suggesting that the grown layer fully covers the sample surface. We have not observed any changes in the diffraction pattern even if with the smallest microdiffraction aperture (≈185 nm), suggesting that the possible domains of each phase are much smaller.

We have compared the diffraction pattern with a series of models using the software LEEDLab,^26^ as shown in Figure 1. A first model, displayed in the upper right quarter, assumes an overlayer composed of two phases: the first, represented in green, is characterized by a monoclinic unit cell with a size of 4.25 Å × 5.41 Å and γ = 95.5° and is rotated by 22° with respect to the Au(111) substrate, while the second (purple) has a monoclinic unit cell of size 2.94 × 3.66 Å^2^ and γ = 91.1°, with a rotation of 30°. The model also accounts for double-scattering events with the substrate.

These 2D crystalline phases were identified by Shpyrko et al.^27^ as surface silicides, with a stoichiometry of Au_4_Si_2_ and AuSi_2_. The authors also observed the formation of a Au_4_Si_8_ silicide, characterized by an orthorhombic 7.386 × 9.386 Å^2^ unit cell with a rotation of 19° with respect to the Au(111) substrate^16,28^ which cannot be seen in the pattern of Figure 1. Interestingly, the formation of these phases was observed at the surface of a molten Au_82_Si_18_ ingot at temperatures above 632 K. In our case, however, the growth temperature is much lower: nevertheless, as evidenced by the diffraction pattern, it leads to the formation of some of these silicide phases as well.

However, not all the diffraction spots can be explained by the current model. The ones indicated by the red arrows in Figure 1 are characterized by a high intensity, indicating a crystalline phase with moderate to high coverage. In the lower-right quarter of Figure 1 we show that these spots can be explained by considering a phase characterized by a rectangular unit cell (4.141 × 7.101 Å^2^, rotated 30° with respect to the Au substrate), which, as we will later show, can be related to a strained silicene layer.

The appearance of 2D hexagonal silicon phases on Au(111) has been already reported.^16,18^ They are, however, characterized by a high number of defects, resulting in deformations of the lattice, which could lead to the appearance of additional diffraction spots. This, in combination with the appearance of diffraction spots due to multiscattering processes, could pose an obstacle to the precise determination of the 2D-Si structure recurring only to the microdiffraction data.

Polarized micro-Raman spectroscopy is an ideal tool for a precise detection and analysis of a hexagonal 2D-Si phase on Au(111). It was shown that a bulk gold–silicide crystalline phase does not give rise to detectable Raman peaks, likely due to the lack of Raman-active vibrational modes,^29^ and is thus incapable of hindering the signal of a silicene phase. 2D silicide phases, instead, could show active Raman modes due to the modified crystalline structure. Recently, it has been shown that after depositing Au on Si(111) and annealing the sample, Raman peaks could be detected, which are associated with the formation of 2D silicides.^30,31^ This is where the study of the polarization dependency makes a difference: the unit cells of the 2D silicides that can be observed in the LEEM pattern and that were previously described in the literature in great detail^27,28^ possess an extremely low symmetry. The two phases that we can detect from the diffraction pattern have a monoclinic unit cell (γ ≠ 90°). Therefore, even if we assume that all the atoms within the unit cell lay in the same plane (zero buckling condition), the highest achievable symmetry, for all of these phases, is represented by point group *C*_2_.

The Raman tensors associated with *C*_2_ structures are of two types:^32^
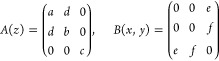
Raman selection rules dictate that in a backscattering geometry B-type phonon modes are always invisible, while A-type modes are visible both in parallel and in crossed polarization configuration.

Differently, if we consider a crystalline structure with a higher symmetry, we can observe polarized Raman modes that are visible in parallel polarization configuration but completely suppressed in crossed polarization. This is true for a hexagonal lattice, even in the case of buckling, and it also holds upon uniaxial deformations, as it happens for black phosphorus, whose structure belongs to point group *D*_2*h*_.^33^

Based on this argumentation, the study of the polarization dependency of the Raman spectrum is extremely important because the identification of a fully polarized Raman mode (invisible in crossed polarization) could not be explainable considering only the presence of the aforementioned silicides phases, but it would be, instead, a clear indication of the presence of a higher symmetry phase.

To perform detailed *ex situ* polarized micro-Raman analysis of the grown 2D silicon layer, it is necessary to protect it from oxidation, which otherwise occurs very rapidly under ambient conditions.^34^ In our previous work, we have presented a method for passivating silicene layers by encapsulating them by few-layer graphene (FLG) flakes.^19^ The passivation, performed directly in UHV, is based on the mechanical exfoliation of graphite on top of the freshly grown silicene layer. The FLG flake forms an exceptional barrier against oxidative species and, thanks to its inert surface, preserves silicene structure unaltered. We have recently extended this procedure to use also few-layer hBN flakes, resulting in a highly transparent and insulating encapsulation layer. It is important to mention that the samples used for the Raman measurements are not the same as the one used for LEEM analysis. However, the growth has been performed under the same conditions. To further confirm that the samples are identical, the ones used for Raman analysis are also investigated by using LEED. The obtained pattern (Figure S1) shows that the structure is identical with the one analyzed by using LEEM. The blurriness of the pattern is caused by a slight mosaicity of the Au(111) substrates that affects the large-area measurements performed by using a classic LEED apparatus.

**Figure 2:**
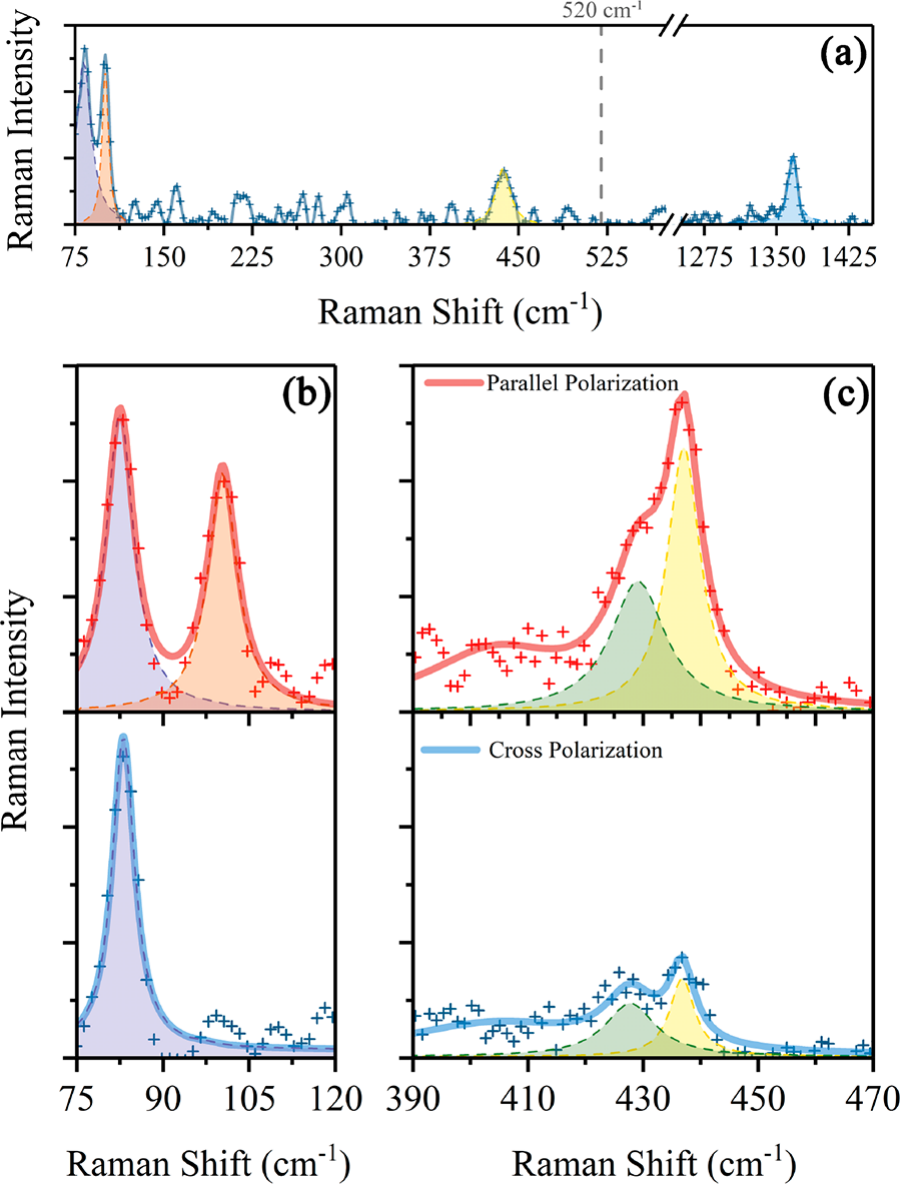
(a) Unpolarized Raman spectrum of the grown layer, protected from oxidation by few-layer hBN. In addition to the well-known vibrational mode for boron nitride at 1360 cm^–1^, three other modes can be distinguished at 83, 100, and 435 cm^–1^. The dashed gray line indicates the position of the well-known 520 cm^–1^ Raman mode of bulk, sp^3^-hybridized Si. (b, c) Polarized Raman spectra of the grown layer encapsulated by few-layer hBN, acquired in backscattering geometry in parallel (top, red line) and cross-polarization (bottom, blue line). Low-frequency modes can be fitted by a single Lorentzian function (purple and orange dashed lines), while the high-frequency peak is fitted with a combination of two Lorentzian functions (green and yellow dashed lines). The two panels (b, c) are separated because the spectra are acquired with different spectrometers.

Figure 2a shows an unpolarized Raman spectrum, acquired in backscattering geometry, 30 min after *in situ* passivation with hBN and subsequent removal from the UHV system. The observed spectrum is identical with the one taken from a sample encapsulated under few-layer graphene flakes (Figure S2), suggesting that the observed Raman peaks are not associated with interlayer vibrational modes, which can be observed in some van der Waals stacks.^35^

The spectrum shows four main peaks at 83, 100, 435, and 1360 cm^–1^, with the last one assigned to the well-known E_2g_ mode of multilayer hBN.^36^ Interestingly, no peak can be found at 520 cm^–1^—shown in more detail in Figure S3—clearly ruling out the presence of detectable crystalline, sp^3^-hybridized, silicon.

It must be noted that at some points on the sample two additional peaks can be recorded between 100 and 435 cm^–1^, positioned at 181 and 304 cm^–1^, both characterized by a low intensity (Figure S4, upper spectrum, red arrows). These peaks, however, are not always detectable and are showing up only at certain locations. A spectrum with a better signal-to-noise ratio, obtained by averaging 30 acquisitions, clearly shows that these peaks are not observable, while the 100 and 435 cm^–1^ peaks still show up at the usual positions, with unchanged width and intensity (Figure S4, lower spectrum). This clearly proves that the two additional peaks are decoupled from the 100 and 435 cm^–1^ ones and belong to a different structure, possibly a signature of the observed silicide phases. The broader shoulders visible in Figure S4 at 260 and 360 cm^–1^ are a possible signature of amorphous structures, as hinted by the much larger FWHM compared to the peaks at 100 and 435 cm^–1^.

To fully characterize the most intense, and always visible vibrational modes, we performed a polarized Raman analysis of the sample. Our results are shown in Figure 2b,c.

In both panels, the upper part shows the signal acquired in parallel polarization configuration, where the polarization direction of the scattered light is parallel to the one of the incident light. The lower half, instead, shows the signal in crossed polarization configuration, where the polarization vectors are normal to each other. The lower frequency region can be analyzed only by using the Alpha 500 Witec setup, albeit with a much worse signal-to-noise ratio and lower spectral resolution. The higher frequency region, instead, is acquired with the Alpha 300 Witec setup, which allows us to obtain a higher spectral resolution and a markedly better signal-to-noise ratio for this spectral region. For this reason, the analysis of the higher frequency peak will be performed by using these data.

First, we can note that the 435 cm^–1^ peak is asymmetric. From the crossed polarization data, it is clear that it is composed of two different, slightly separated modes. A least-squares fitting process allows us to model the modes with Lorentzian functions. The results show that the two peaks are centered at 429 ± 2 and 437.0 ± 0.5 cm^–1^, with a FWHM of 11 ± 4 and 7 ± 2 cm^–1^, respectively.

An additional broad peak centered around 410 cm^–1^ is related either to phonon confinement effects or to a vibrational mode that was observed also for silicene on Ag(111) and, in that case, assigned to a second-order phonon mode.^37^

The low-frequency modes can be fitted with a single Lorentzian function, centered at 82.8 ± 0.2 and 100.4 ± 0.2 cm^–1^, with a FWHM of 5.0 ± 0.5 and 6.5 ± 0.5 cm^–1^.

No difference can be observed when fitting the peaks of the spectrum related to silicene capped under few-layer graphene flakes, both regarding their positions and the FWHM values. The intensities of the peaks in the graphene-capped sample are lower as the few-layer graphene flake is less transparent than the hBN one.

Clearly, the Raman peaks at 100.4 and at 437 cm^–1^ show a strong polarization dependency. Particularly the lower frequency mode is completely suppressed in crossed polarization configuration, with a reduced intensity within the noise level. For the reasons explained earlier, this fully polarized Raman peak is a clear indication of the existence of a high symmetry phase in the sample, as it cannot be generated by the silicide phases. These peaks appear to be strongly red-shifted, if compared to the Raman peaks predicted for freestanding silicene, where the modes are expected at 175 and 566 cm^–1^,^38−40^ or to the ones observed for silicene on Ag(111), where the out-of-plane mode is centered at 216 cm^–1^ and in-plane modes are detected at 515 cm^–1^.^37,41,42^ This denotes a substantial structural modification that is likely induced by a rather strong interaction with the substrate.

It must be noted that a softening (i.e., red-shift) of the in-plane vibrational modes implies longer Si–Si bonds. Notably, it was recently observed that a 2D hexagonal Si phase on Au(111) is characterized by much larger lattice constant (4.1 Å, compared to 3.81 Å for strain-free silicene).^18^

**Figure 3:**
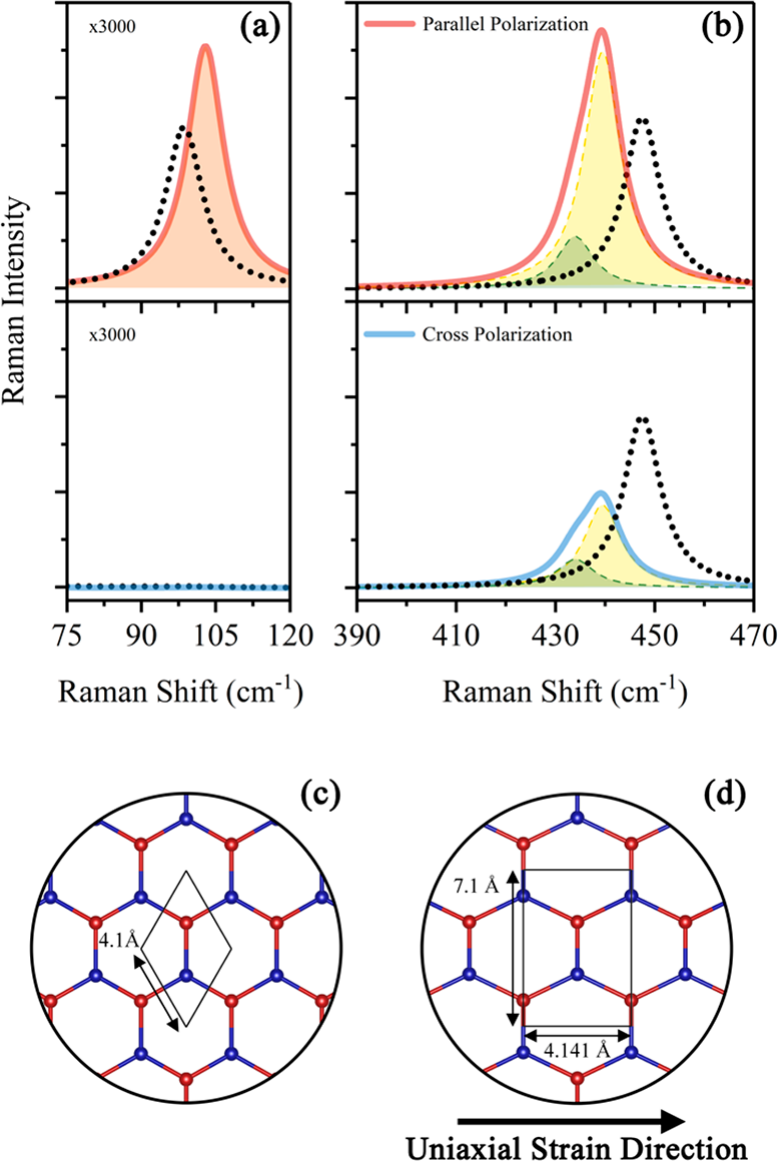
(a, b) Simulated Raman spectra in parallel and cross-polarization for biaxially strained (7.6%) silicene (black dotted line) and for biaxially strained silicene with a superimposed additional uniaxial strain of 1% (solid red and blue lines). The doubly degenerate in-plane mode splits into two separated peaks (green and yellow dashed lines). (c) Simulated structure of biaxially strained silicene. The rhomboidal unit cell (black solid line) edge length is 4.1 Å, with γ = 60°. Red atoms are buckled along the out-of-plane direction by 0.237 Å. (d) Simulated structure of biaxially strained silicene with a superimposed additional uniaxial strain. For clarity and visualization purpose, the image represents a lattice deformed by a uniaxial strain of 20%. The unit cell dimensions (4.141 × 7.1 Å^2^) are relative to the simulated 1% uniaxial strain.

Thus, to confirm this hypothesis of a strained 2D silicene phase, we performed ab initio calculations of the vibrational modes of a silicene structure with a lattice constant of 4.1 Å. At first, we fully characterized a freestanding layer of silicene, obtaining a lattice parameter of 3.810 Å, a buckling of 0.425 Å, and Γ-point phonons of 175 and 566 cm^–1^. Next, we reoptimized the atomic positions, constraining the lattice parameter of silicene to the value of 4.1 Å, corresponding to a biaxial tensile strain of 7.6%. In these highly strained conditions, the buckling is strongly reduced to 0.237 Å, although, differently from the flat geometry reported in ref (18), it does not vanish. The relative phonon dispersion plots are shown in Figure S5. The computed Raman spectra, in both the parallel and crossed polarization geometry, are shown as dashed black lines in Figure 3a,b. The simulated structure that gives rise to these spectra is shown in Figure 3c, described by a rhomboidal unit cell characterized by an edge length of 4.1 Å and γ = 60°.

The positions of the simulated modes are in good agreement with the experimental data, with a calculated out-of-plane mode centered at 98.6 cm^–1^ and the two degenerate in-plane modes centered at 447.3 cm^–1^. The experimentally observed mode centered at 83 cm^–1^, instead, does not find any match within the proposed model and could be related to edge- or defect-activated vibrational modes.

Additionally, to verify whether such a structure can be accommodated on gold, we placed the strained silicene layer on top of an ideally terminated Au(111) surface—something that we could not perform when calculating the Raman response, due to computational limitations—and relaxed the atomic positions until all forces were lower than 0.02 eV/Å. A stable configuration is indeed readily obtained, supporting the picture that such a biaxially strained silicene structure can be stabilized by the Au(111) substrate.

It must be noted that the experimental data show a splitting of the in-plane modes, with the 437 cm^–1^ peak characterized by a much stronger polarization dependence. The theoretical calculations, however, predict that the two in-plane modes are degenerate, with no change in intensity between parallel and crossed polarization configurations, as expected given the symmetry of the lattice. This loss of degeneracy could be caused by a small deviation from the perfect hexagonal lattice due to an additional uniaxial distortion of the structure, similarly to what can be observed in uniaxially strained graphene.^43^ This could be induced by the peculiar properties of Au(111), which is known to form a complex surface reconstruction—the herringbone reconstruction—characterized by an uniaxial deformation along one of the ⟨11̅0⟩ directions.^44^ For this reason, we additionally imposed a small uniaxial strain (1%) to the previously simulated structure along the zigzag direction (see Figure 3d) and monitored the Raman response. Under the additional strain the vibrational modes significantly change, improving the agreement with the experimental data. The out-of-plane mode exhibits a small blue-shift, up to 102.9 cm^–1^. The in-plane vibrational modes get red-shifted and split up in two distinct peaks, as the degeneration is lifted, at 433.9 and 439.5 cm^–1^. The simulated spectrum obtained by adding 1% additional strain exhibits also a polarization dependency, with the higher frequency mode showing a more pronounced intensity reduction in crossed polarization configuration as compared to the lower frequency one, similar to what can be observed experimentally.

Despite showing a good agreement regarding the frequency of the peaks and the polarization dependence, the simulations predict a much lower intensity for the out-of-plane vibrational mode (which is magnified ∼3000 times in Figure 3a) compared to the in-plane ones. We attribute this discrepancy to the presence of the substrate, not accounted for in the theoretical calculations. Au(111)/mica is a SERS-active substrate,^45^ and it is well-known that the amplification factors of different vibrational modes can noticeably vary.^46^ Such an amplification is expected to be particularly effective for an out-of-plane vibration, which could couple well with the metallic substrate.

Figure 3d depicts how uniaxial strain modifies the lattice of the biaxially strained silicene shown in Figure 3c. For a better visualization of the structural modifications, the image shows the effects of 20% uniaxial strain. The modified structure can be optimally described by choosing a rectangular unit cell, with dimensions of 4.141 × 7.1 Å^2^, which possesses all the symmetry properties of the deformed lattice. The corresponding diffraction pattern, represented by black circles in the lower right quarter of Figure 1, explains the intense spots, indicated by red arrows, that were not addressed by the silicide-only model.

Based on these results, Au(111)/mica is clearly a promising platform to study the effects of strain on silicene. The effects of biaxial tensile strain on the silicene electronic structure have already been explored in the literature by using first-principles calculations. The results of the calculations show that despite the huge mechanical deformation, the Dirac cone is preserved.^47,48^ For high biaxial strain levels, the tip of the Dirac cone shifts above the Fermi energy, inducing the formation of hole-doped Dirac states. The level of biaxial strain that induces this transition varies among different studies, ranging from 6%^47^ to 8%.^48^ Au(111) could represent a perfect platform for the investigation of such effect, as the strain level of the detected silicene phase is very close to those values.

Future investigations will focus on probing the electronic structure of silicene grown on Au(111)/mica, searching for a confirmation of its Dirac nature.

## 4 Conclusions

We have presented a study of the vibrational properties of a silicene layer epitaxially grown on Au(111). The diffraction pattern can be explained by a combination of two surface silicide phases and a strained silicene-derived phase.

A passivation step, obtained by encapsulating the grown layer under few-layer hBN or graphene flakes, allowed us to perform polarized Raman spectroscopy. The observed vibrational spectrum is characterized by polarization-dependent peaks that, for symmetry reasons, cannot be related to the silicide structures. Hence, the detected modes are a clear indication for the presence of a higher symmetry phase. Using first-principles calculations, we identified this phase as a highly (>7%) biaxially strained silicene phase, slightly deformed along one direction.

The present work gives clear experimental evidence on the existence of a stable, highly strained silicene phase on Au(111), indicating a favorable platform for the investigation of the effects of strain on this promising 2D material.

## Supporting Information Available

S1: additional LEED pattern; S2: additional Raman spectrum of silicene/Au(111) encapsulated under a few-layer graphene flake; S3: Raman signal near sp3-silicon peak position; S4: additional Raman spectrum showing low-intensity peaks; S5: phonon dispersion curves (PDF)
