# Supplementary material for: Highly Biaxially Strained Silicene on Au(111)
Source: J Phys Chem C Nanomater Interfaces. 2021 May 4;125(18):9973–80. doi: 10.1021/acs.jpcc.0c11033 (PMC8154839; doi:10.1021/acs.jpcc.0c11033)
Supplement: Supplementary file 1 — jp0c11033_si_001.pdf [file jp0c11033_si_001.pdf]

Supporting Information

Highly biaxially strained silicene on Au(111)

Daniele Nazzari†, Jakob Genser†, Viktoria Ritter†, Ole Bethge‡, Emmerich Bertagnolli†, Georg Ramer§, Bernhard Lendl§, Kenji Watanabe$, Takashi Taniguchi°, Riccardo Rurali&, Miroslav Kolíbal#,£ and Alois Lugstein†*

† Institute of Solid State Electronics, Technische Universität Wien, Gußhausstraße 25-25a, 1040 Vienna, Austria

‡ Infineon Technologies Austria AG, Siemensstraße 2, 9500 Villach, Austria

§ Institute of Chemical Technologies and Analytics, Technische Universität Wien, Getreidemarkt 9, 1060 Vienna, Austria

$ Research Center for Functional Materials, National Institute for Materials Science, 1-1 Namiki, Tsukuba 305-0044, Japan

° International Center for Materials Nanoarchitectonics, National Institute for Materials Science, 1-1 Namiki, Tsukuba 305-0044, Japan

& Institut de Ciència de Materials de Barcelona, ICMAB-CSIC, Campus UAB, 08193 Bellaterra, Spain

Institute of Physical Engineering, Brno University of Technology, Technická 2, 616 69 Brno, Czech Republic

£ CEITEC BUT, Brno University of Technology, Purkyňova 123, 612 00 Brno, Czech Republic

*E-mail: alois.lugstein@tuwien.ac.at.

Number of pages: 4

Number of figures: 5

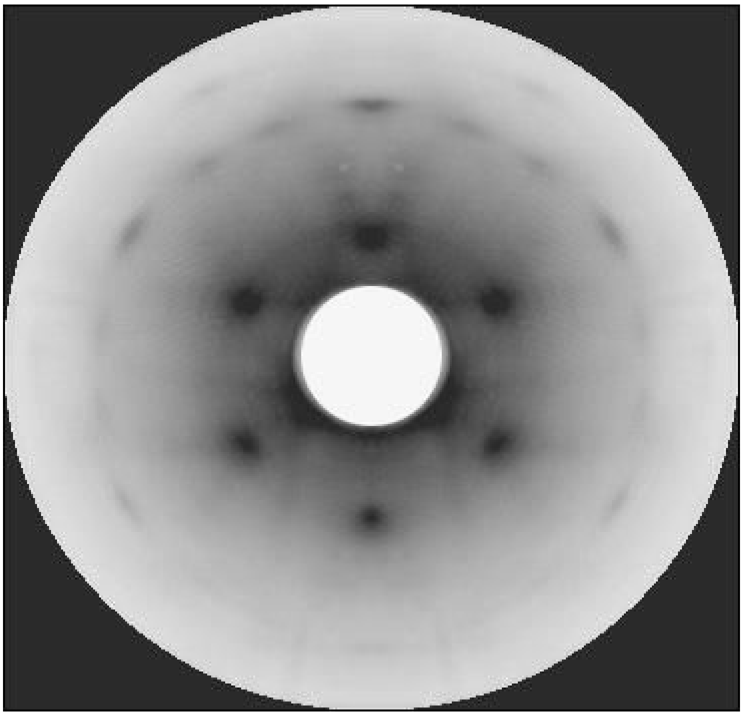

Fig. S1 – LEED pattern of 1 ML of silicene grown on Au(111) acquired at an electron energy of 35 eV.

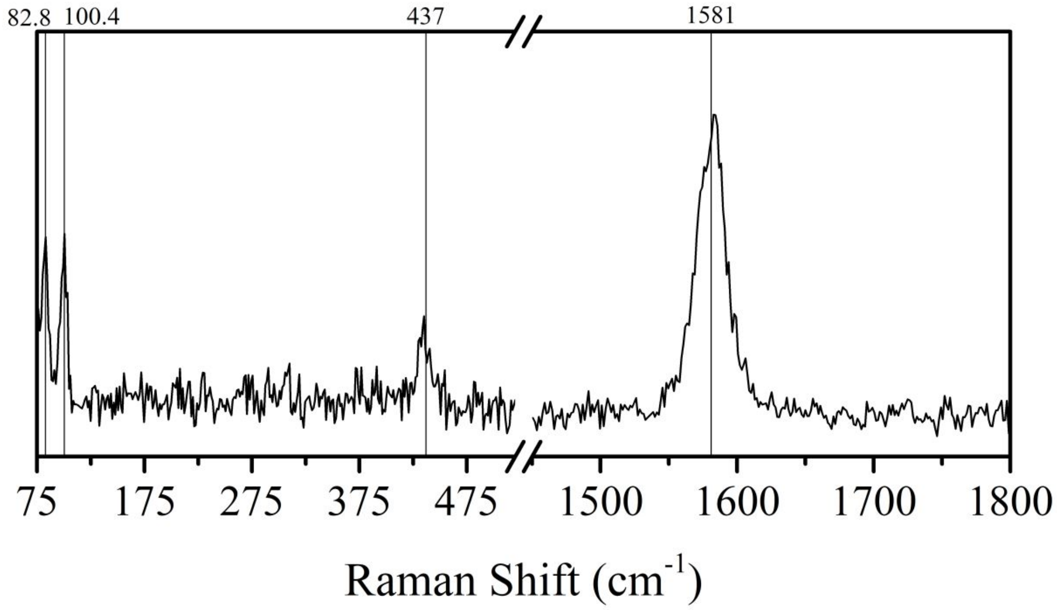

Fig. S2 - Unpolarized Raman spectrum of 1 ML of silicene grown on Au(111) and encapsulated under a few-layers graphene flake. The silicene peak positions are unchanged if compared to the hBN-encapsulated layer. The peak centered at 1581 cm^{-1} is the characteristic G peak for multilayer graphene.

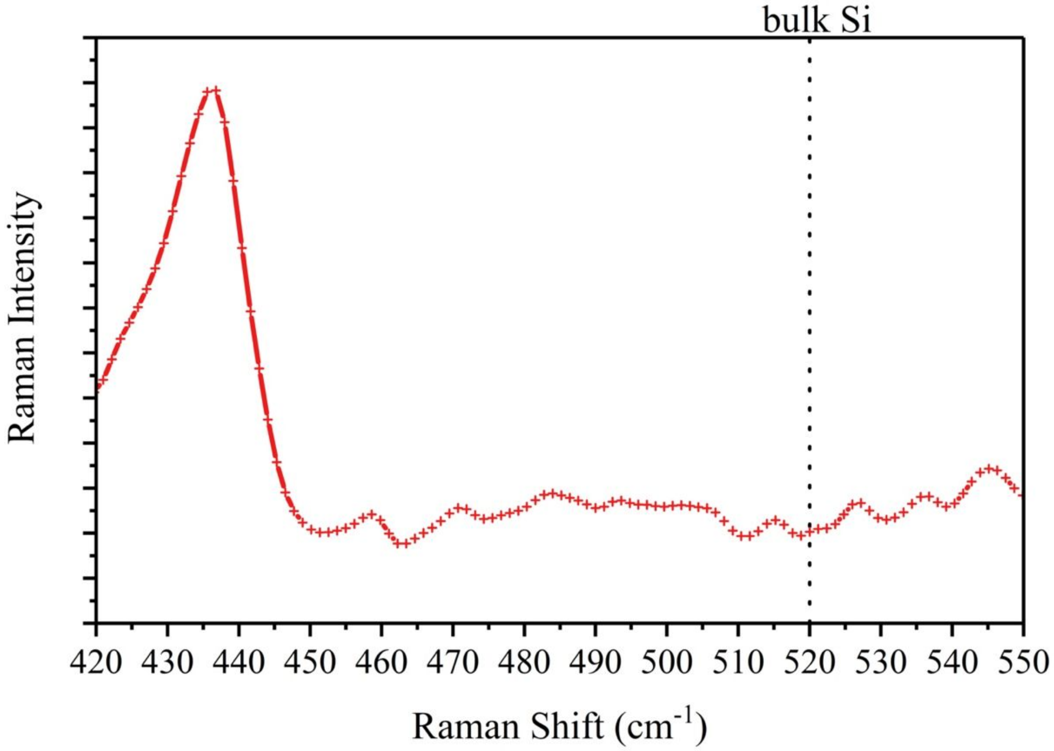

Fig. S3 – Unpolarized Raman spectrum in the spectral region near 520 cm^{-1}. No bulk silicon (sp^3 hybridized) response can be detected.

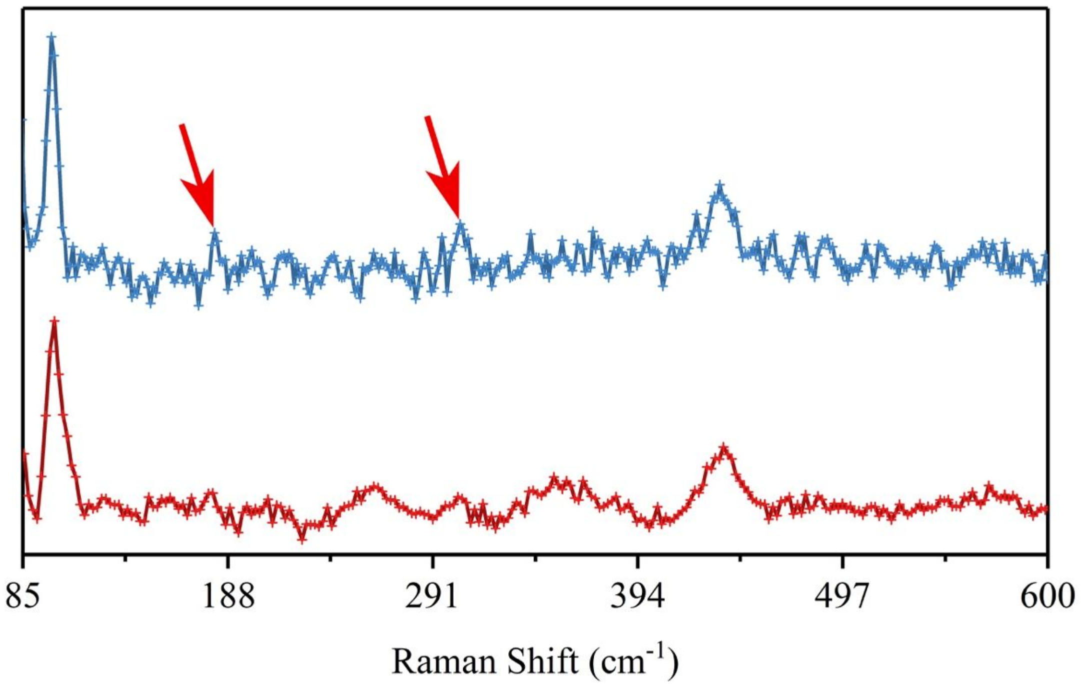

Fig. S4 – Unpolarized Raman spectra of 1 ML of silicene grown on Au(111) and encapsulated under a few-layers hBN flake. The upper spectrum (blue) shows the appearance of low-intensity peaks located at 181 cm^{-1} and 304 cm^{-1}, indicated by red arrows. These peaks are not always visible as shown in the lower spectrum (red), thus decoupled from the high intensity peaks located at 100 cm^{-1} and 435 cm^{-1}. The red spectrum is the result of 30 spectra averaged together (collected at the same point on the sample)

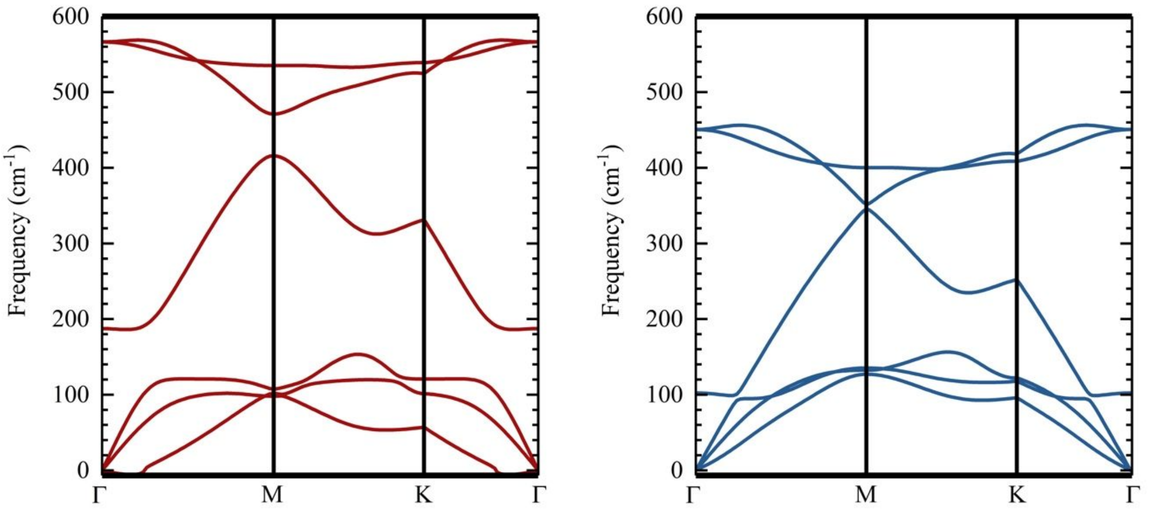

Fig. S5 – Phonon dispersion curves calculated along the G->M->K-> G path for the freestanding (left, red) and biaxially strained (right, blue) silicene, using the finite displacement method in a 7x7 supercell.
